# Renal HIV Expression Is Unaffected by Serum LPS Levels in an HIV Transgenic Mouse Model of LPS Induced Kidney Injury

**DOI:** 10.1371/journal.pone.0020688

**Published:** 2011-06-16

**Authors:** Jeremy S. Leventhal, Zygimantas Alsauskas, Alexandra Snyder, Pengfei Gong, Bin Wang, Vivette D'Agati, Michael J. Ross

**Affiliations:** 1 Division of Nephrology, Department of Medicine, Mount Sinai School of Medicine, New York City, New York, United States of America; 2 Division of Nephrology, James J. Peters Bronx VA Medical Center, Bronx, New York, United States of America; 3 Division of Nephrology, University of Louisville, Louisville, Kentucky, United States of America; 4 Department of Medicine, Mount Sinai School of Medicine, New York City, New York, United States of America; 5 Department of Pathology, Columbia University Medical Center, New York, New York, United States of America; University of California, San Francisco, United States of America

## Abstract

Acute kidney injury (AKI) is associated with increased rates of mortality. For unknown reasons, HIV infected individuals have a higher risk of AKI than uninfected persons. We tested our hypothesis that increased circulating LPS increases renal expression of HIV and that HIV transgenic (Tg26) mice have increased susceptibility to AKI. Tg26 mice harbor an HIV transgene encoding all HIV genes except *gag* and *pol*, and develop a phenotype analogous to HIVAN. Mice were used at 4–6 weeks of age before the onset of gross renal disease. Mice were injected i.p. with LPS or sterile saline. Renal function, tubular injury, cytokine expression, and HIV transcription were evaluated in Tg26 and wild type (WT) mice. LPS injection induced a median 60.1-fold increase in HIV expression in spleen but no change in kidney. There was no significant difference in renal function, cytokine expression, or tubular injury scores at baseline or 24 hours after LPS injection. HIV transcription was also analyzed in vitro using a human renal tubular epithelial cell (RTEC) line. HIV transcription increased minimally in human RTEC, by 1.47 fold, 48 hours after LPS exposure. We conclude that Tg26 mice do not increase HIV expression or have increased susceptibility to LPS induced AKI. The increased risk of AKI in HIV infected patients is not mediated via increased renal expression of HIV in the setting of sepsis. Moreover, renal regulation of HIV transcription is different to that in the spleen.

## Introduction

Recent data has identified HIV infected patients as a group predisposed to acute kidney injury (AKI). A study by Wyatt et al. analyzing billing codes for patients in New York state for the years 1995 and 2003, before and after the introduction of antiretroviral therapy, revealed a risk for AKI in hospitalized HIV patients 2.82 times higher than their uninfected counterparts[1]. Furthermore, HIV infected patients with AKI had a mortality risk 5.83 times higher than patients without renal failure[1]. Importantly, as opposed to the renal syndrome of HIV associated nephropathy (HIVAN), black race was not an identified risk factor for AKI in the HIV patients. An increased risk for AKI has also been observed in both hospitalized and ambulatory HIV infected patient populations[2], [3]. This phenomenon is now well described in the clinical literature yet lacks a mechanistic explanation.

Over half of the AKI events in an ambulatory HIV patient population were related to infection[3]. Much research has been conducted studying the effects of enteric bacterial translocation, reflected in elevated serum lipopolysaccharide (LPS) levels, on HIV progression and immune activation. In general, these studies have found that higher circulating LPS levels are associated with increased viral replication and a more advanced disease state[4]; Brenchley et al., 2006, Nat Med, 12, 1365–71}. The relationship between serum LPS and the predisposition of HIV patients to AKI has not been investigated.

Elevated serum LPS can be seen during bacterial sepsis, and septic AKI can be modeled in animals by intraperitoneal injection of LPS. LPS stimulates Toll-like receptor 4 (TLR4) of the innate immune system. The resulting release of cytokines, mediated through nuclear factor kappaB (NF-kB) activation[5], contributes to AKI and is represented by increases in serum urea nitrogen (SUN) and tubular injury [6].

First demonstrated in monocytes, NF-κB is also a necessary transcription factor for HIV replication and its activation results in HIV gene expression[7]. Subsequently, a relationship between TLR4 activation and HIV transcription was demonstrated when incubation of exposed human dermal microvessel endothelial cells (HMEC) with LPS increased the activation of an HIV LTR luciferase reporter construct[8]. Notably, this effect was abrogated by cotransfection of dominant negative TLR4/NF-κB signaling molecules[8], further supporting a role for LPS signaling through TLR4 in activation of HIV transcription. We therefore hypothesized that LPS would also increase HIV transcription in renal tubular epithelial cells (RTEC).

Whereas increased HIV transcription and plasma viral RNA is recognized to occur in the setting of bacterial infection, research on specific tissue transcriptional responses to LPS have been conflicting[9], [10]. Several studies have found that the activating role of LPS described in HMEC[8]. LPS incubation does not increase HIV transcription in macrophages because of interferon-induced suppression[11] that, conversely, decreases HIV replication. Another study found that HIV transcription, measured with a pNL4-3-luc reporter construct, failed to increase after stimulation with LPS[12]. Notably, the experiment was conducted using transfected HEK293T cells, a cell line originally derived from embryonic kidney.

The effect of LPS on renal HIV transcription, and its subsequent role in the pathogenesis of septic AKI has not been investigated. In this paper, we studied whether LPS induces increased renal HIV transcription and if renal HIV expression leads to exaggerated inflammatory cytokine production, exacerbated tubular injury and worsened renal function.

## Methods

### Ethics Statement

Biopsy tissue, from which the RT3 cell line was derived, was collected at the time of diagnostic renal biopsy after written consent was obtained under a protocol approved by the Mount Sinai Institutional Review Board. All animal studies were performed under a protocol (#07-1224) approved by the Mount Sinai Institutional Animal Care and Utilization Committee (IACUC) that adheres to NIH guidelines of animal care.

### HIV Transgenic Mice

The “Tg26” HIV-transgenic model of HIVAN has been extensively characterized. [13]–[15] These mice harbor an HIV provirus with deletions spanning the *gag* and *pol* genes that are expressed under control of the endogenous HIV LTR promoter. Heterozygous Tg26 mice develop disease closely mimicking the human HIVAN phenotype several months after birth. We used four to six week-old mice in these studies, an age at which Tg26 develop proteinuria but not renal failure or histologic renal disease.

### Animal Studies

Four to six week-old heterozygous Tg26 mice and/or wild-type FVB/N (WT) littermates (controls) were weighed and administered 20 mg/kg of *Escherichia coli* 0111:B4 standard preparation LPS (Invivogen) or sterile saline by intraperitoneal (*i.p.)* injection. Blood was drawn from mice immediately prior to injections and immediately prior to sacrifice 24 hours later. Serum urea nitrogen (SUN) concentrations were measured via photometric assay on a Prochem V apparatus (Drew Scientific). At the time of sacrifice, the right kidney was placed in 4% paraformaldehyde/PBS and subsequently embedded in paraffin and the left was snap frozen in liquid nitrogen and stored at −80 Celsius.

### Quantitative real time PCR

Small slices (∼100 mg) of snap frozen cortex from kidneys of injected mice were homogenized in TRI REAGENT (Molecular Research Center) with a handheld motorized pestle (Kontes). RNA was isolated and quantified using a Nanodrop spectrophotometer (Thermo Scientific). 5 ug of total RNA was reverse transcribed using SuperScript III First-Strand Synthesis System (Invitrogen). cDNA was analyzed by quantitative real time PCR (qPCR) at the Mount Sinai Quantitative PCR Shared Research Facility on an ABI PRISM 7900HT Sequence Detection System using the following parameters: 15 minutes at 94C, 1 minute at 58C, 1 minute at 72C for 40 cycles. Primers for amplification of cyclophilin A (housekeeping control), MCP-1, TNF-a, CXCL5, ICAM-1, IL-6, and IL-10 have been previously published [16]–[19]. Amplification of HIV *tat/rev* and *tat/rev/nef* transcripts was performed with previously validated primer sets[20]. Primers for IFN-a (forward) 5′ GGTGGAGGTCATTGCAGAAT 3′, (reverse) 5′ ATGCCCAGCAGATCAAGAAG 3′, IFN-b (forward) 5′ CCCAGTGCTGGAGAAATTGT 3′, (reverse) 5′ CCCTATGGAGATGACGGAGA 3′, murine Aquaporin 1 (forward) 5′ AGGCTTCAATTACCCACTGGA 3′, (reverse) 5′ AGGCTTCAATTACCCACTGGA 3′, murine Cytokeratin 18 (forward) 5′ CAGCCAGCGTCTATGCAGG 3′, (reverese) 5′ CTTTCTCGGTCTGGATTCCAC 3′, murine E-cadherin (forward) 5′ CAGGTCTCCTCATGGCTTTGC 3′, (reverse) 5′ CTTCCGAAAAGAAGGCTGTCC 3′, murine Tamm-Horsfall (forward) 5′ GACCTGGATGCTGCTGGTAAT 3′, (reverse) 5′ GTGGCGTTGTTGTGGCATTC 3′, murine von Willebrand's Factor (forward) 5′ CTTCTGTACGCCTCAGCTATG 3′, (reverse) 5′ GCCGTTGTAATTCCCACACAAG 3′, human Aquaporin 1 (forward) 5′ TTAACCCTGCTCGGTCCTTTG 3′, (reverse) 5′ AGTCGTAGATGAGTACAGCCAG 3′, human Cytokeratin 18 (forward) 5′ ACAATGCCCGCATCGTTCT 3′, (reverse) 5′ GGATGTCGTTCTCCACAGACT 3′, human E-cadherin (forward) 5′ CCCACCACGTACAAGGGTC 3′, (reverse) 5′ ATGCCATCGTTGTTCACTGGA 3′, human Tamm-Horsfall (forward) 5′ CCGGCGGCTACTACGTCTA 3′, (reverse) 5′ TGCCATCTGCCATTATTCGATTT 3′, and human von Willebrand's Factor (forward) 5′ CCTGCCGCTCTCTCTCTTAC 3′, (reverse) 5′ AGCACATGGTGTGATGGTCTG 3′ were designed using Primer Bank (http://pga.mgh.harvard.edu/primerbank/).

### Statistics

Gene expression was calculated using the 2^(-DDCT)^ method [21], [22]. In short, 2^(-DCT)^ was calculated by first averaging the DC_T_ (C_T_Target gene-C_T_Control gene) for replicates of each individual sample and then calculating the mean of the averaged sample DC_T_ values. Changes in expression were calculated by dividing the average DC_T_ for each gene in an experimental group (LPS-injected Tg26, WT, and saline-injected Tg26) by the DC_T_ of the control group (saline-injected WT). Changes in expression for an individual animal were obtained similarly. Because the individual fold change data was not distributed normally, differences in median fold change were first compared using a Kruskal-Wallis test. Subsequent pair-wise comparisons were performed with a Mann-Whitney Test to detect significant differences between individual groups.

Acute Tubular Necrosis (ATN) scores of H&E stained paraffin embedded sections were similarly analyzed by first analyzing for significant differences between both control and LPS injected groups using a Kruskal-Wallis test. Pair-wise comparisons of individual group ATN scores were performed using the Mann-Whitney Test. Statistically significant differences were defined by two-sided p<0.05.

### Cell culture

RT3 cells, a human RTEC line, were grown from a human biopsy sample collected in protocol approved by the Mount Sinai Institutional Review Board, derived according to protocol as previously described[23]. RT3 cells were conditionally immortalized with pseudotyped lentivirus expressing temperature sensitive large T-antigen as described previously [23]. Cells were grown in media, modified from previous publications, consisting of Ham's F12/DMEM with 1% fetal bovine serum (Invitrogen), 10 ng/ml EGF (Sigma), 5 ug/ml transferrin (Sigma), 5 ug/ml insulin (Sigma), 50 nmol/L selenium (MP Biomedical), 0.05 mmol/L Hydrocortisone (Sigma), 5 pmol/L tri-iodothryronine (Sigma), 1 ng/ml Prostaglandin E1 MP Biomedical, 50 U/ml penicillin and 50 ug/ml streptomycin [24], [25].

To characterize RTEC cells, RNA was harvested using Trizol (Molecular Research Center, Inc) per manufacturers directions and expression of tubular epithelial markers Aquaporin-1, Cytokeratin 18, and Tamm-Horsfall protein compared to endothelial cell von Willebrand's Factor was analyzed by qPCR as described above in “Quantitative real time PCR”. For studies assessing the response to LPS incubation, confluent cells were incubated in media supplemented with 1 mg/ml of LPS for predetermined time points. RNA was then harvested and analyzed for expression of HIV using primers described above. All data presented was obtained from four independent experiments.

### HIV-1 Transduction

To optimize the efficiency of HIV transduction, we infected RT3 cells with VSV-NL4-3:DG/P-EGFP, a lentiviral vector that expresses the same parental *gag/pol*-deleted HIV provirus used to create Tg26 mice. This vector also expresses enhanced green fluorescent protein (EGFP) and is pseudotyped with the vesicular stomatitis virus glycoprotein to broaden its tropism. We used this virus to transduce human RTEC as described previously[19]. RT3 cells were incubated with viral supernatants for six hours and then incubated in fresh media for an additional 96 hours before being used in further experiments.

### Renal histology

Immediately following sacrifice, the right kidney was stripped of its capsule and immersion fixed in a 4% paraformaldehyde solution. Kidneys were paraffin-embedded, cut into 3 micron sagittal sections, and stained with hematoxylin and eosin. The slides were assigned an acute tubular necrosis (ATN) score by a blinded renal pathologist (Vivette D'Agati, Columbia University). Each kidney cross section was evaluated over its entire area under 20x and 40x magnifications for histologic evidence of tubular single cell necrosis. The percentage of renal cortical area exhibiting tubular epithelial necrosis was scored as follows: 0, none; 1, 10–25%; 2, 26–50%; 3>50%.

## Results

### HIV-transgenic mice are not predisposed to LPS-induced AKI

To determine whether HIV-transgenic mice (Tg26) demonstrate increased susceptibility to LPS-induced AKI, we injected Tg26 mice and WT littermates with LPS. We used four to six week-old mice, an age at which Tg26 mice in our colony do not typically have histological renal disease or renal failure, to avoid the confounding effect that preexisting chronic kidney disease would have upon susceptibility to AKI.

**Figure 1:**
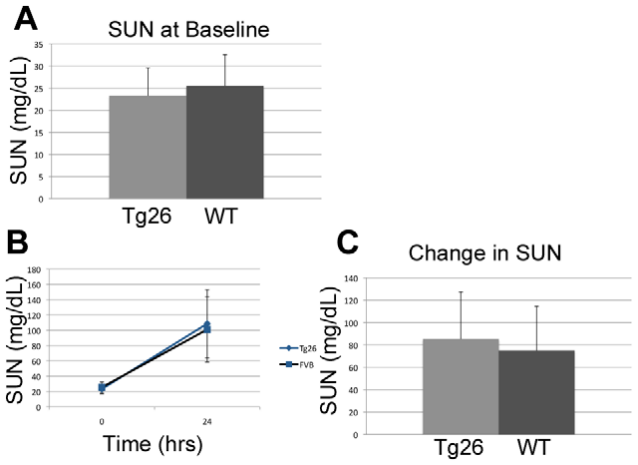
HIV-transgenic mice are not predisposed to LPS-induced AKI. Blood was drawn from Tg26 mice and wild-type littermates prior to injection with LPS. (A) Comparison of baseline SUN demonstrates no statistical difference between the two groups (P = 0.29). (B)Twenty-four hours after LPS injection, similar increases in SUN were seen in both groups. (C) The rise of SUN in Tg26 and WT mice, 85.4 and 75.1 mg/dL, respectively, was not statistically significant (P = 0.41).

**Figure 2:**
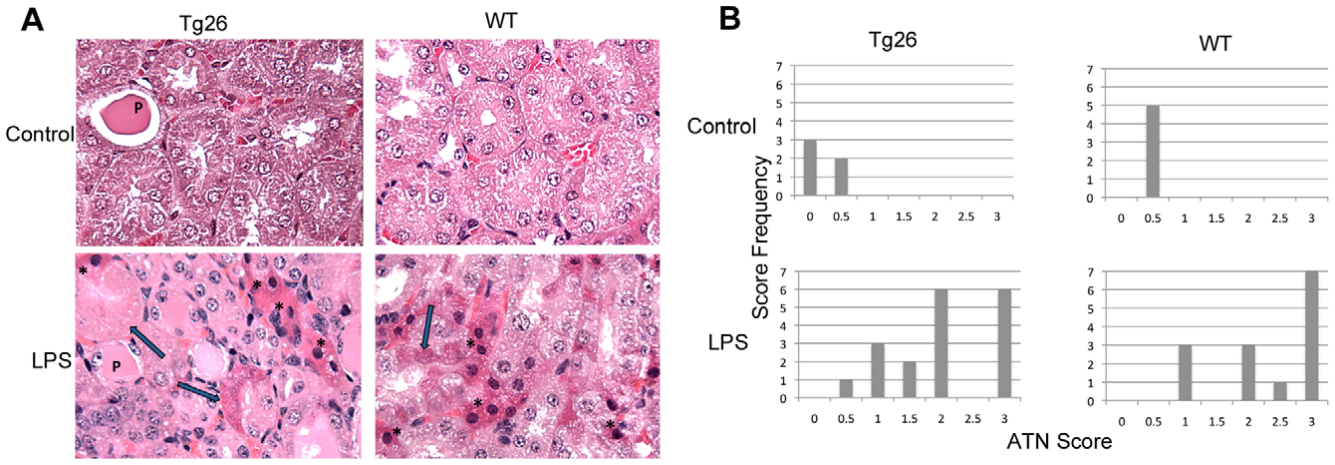
LPS causes similar tubular injury in HIV transgenic and wild type mice. (A) Hematoxylin and eosin stained sections from Tg26 and wild type (WT) mice demonstrated features of acute tubular cell necrosis (ATN) including nuclear condensation (*), cytoplasmic swelling and hypereosinophilia, and focal loss of nuclei (arrow) only in LPS injected samples (bottom panels, x400) and not in control injected mice (top panels, x400). Proteinaceous casts (P), a feature of murine HIVAN, were seen in the tubules of both control and LPS injected Tg26 mice. (B) Tubular damage in LPS injected mice resulted in significantly higher ATN scores compared to control mice (p<.001) but not when compared to each other (p = .60).

There was no difference in the baseline serum urea nitrogen (SUN) of Tg26 and WT mice (Figure 1A). Twenty-four hours after LPS injection, SUN increased in both WT and Tg26 mice (Figure 1B) with 19 of 24 Tg26 and 16 of 20 wild type mice developing a 2-fold or greater increase in SUN. However, the differences between the rise of SUN in both groups, 85.4 mg/dL in Tg26 and 75.1 mg/dL in WT mice, was not statistically significant (Figure 1C, p = 0.41). Single cell tubular epithelial necrosis was noted in all LPS injected samples (Figure 2A) and acute tubular necrosis (ATN) scores were significantly higher in LPS injected samples (Figure 2B). However, there was no significant difference in the ATN scores between LPS injected WT and Tg26 mice (p = 0.60).

### LPS exposure increases HIV transcription in spleen but not in kidney tissue

Since LPS is known to increase expression of NF-κB-regulated genes, and NF-κB is an important HIV transcription factor, we studied the effect of LPS on increased HIV transcription in Tg26 mice *in vivo*. Four to six week old Tg26 mice were injected *i.p.* with LPS or saline. 24 hours after injection, renal HIV transcription levels in LPS-injected mice did not significantly differ from saline-injected mice (p = 0.27).

LPS has been shown to increase splenic HIV transcription in another HIV-transgenic mouse model [26]. We therefore studied whether LPS injection increased splenic HIV transcription in Tg26 mice and whether the change in splenic HIV transcription differed from kidney. In contrast to kidneys, spleens from LPS-injected Tg26 mice had a more than 60.1-fold increase in HIV transcription compared to spleens of saline-injected Tg26 controls (p<0.028).

### Effect of LPS exposure upon HIV transcription in RTEC in vitro

**Figure 3:**
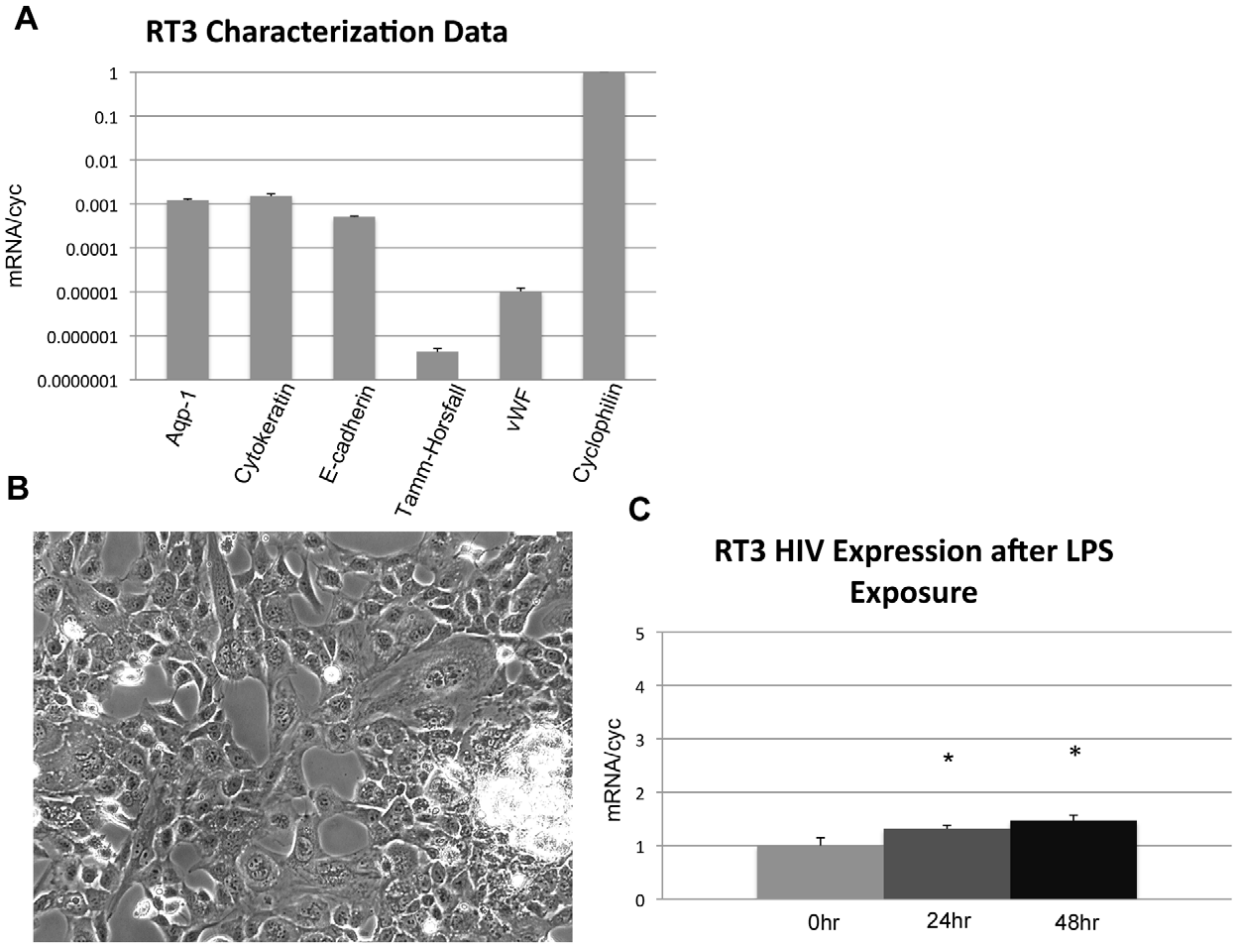
LPS induces a minimal increase in HIV transcription in an infected human RTEC cell line. (A) qPCR analysis of RT3 RNA demonstrated expression of the epithelial markers cytokeratin ande-cadherin and the proximal tubular marker aquaporin-1. RT3 cells did not express distal tubular marker Tamm-Horsfall protein or von Willebrand's factor (endothelial marker). (B) The tubular cell line RT3 displays tubular cell morphology, with polygonal/refractile appearance, and tendency to form characteristic domes when overgrown. (C) The human RT3 cell line was transduced with a *gag/pol*-deleted HIV lentiviral vector and incubated with LPS four days later. LPS induced a minimal, but significant (*), increase in HIV RNA that was 1.32 and 1.47-fold after 24 and 48 hours, respectively (p<0.05).

We then examined the effect of LPS on RTEC HIV transcription *in vitro*. Since murine cells lack factors necessary to support optimal HIV transcription[27], [28] we studied LPS-induced HIV transcription in human RTEC. Infection of human RTEC with wild-type HIV virus is inefficient [29]. Therefore, in these studies we infected the human RTEC line, RT3, with a VSV-pseudotyped lentiviral replication-incompetent HIV-1 vector (VSV-NL4-3:DG/P-EGFP) that has been used in previous studies modeling the RTEC response to HIV [19], [23], [30]. RT3 cells were derived from a patient biopsy and display normal RTEC morphology and express typical proximal RTEC markers (Figure 3A and 3B). Three days after infection, RT3 cells were exposed to 1 *m*g/ml LPS and HIV RNA levels were evaluated by qPCR. Two days after exposure, LPS induced a minimal, though statistically significant 1.32 and 1.47-fold (p<0.05) increase in HIV transcription, respectively (Figure 3C).

### Effects of LPS injection on renal cytokine expression HIV transgenic and wild type mice

**Figure 4:**
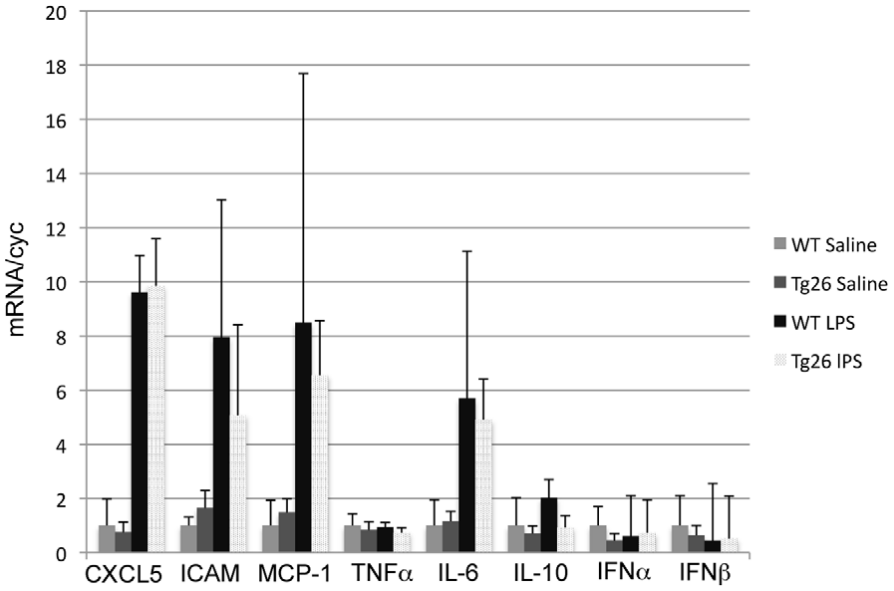
Cytokine analysis of saline and LPS injected animals. qPCR analysis was performed using RNA from kidneys of Tg26 and wild type mice injected with LPS or saline. The expression of several mediators of inflammation and modifiers of viral transcription were examined. CXCL5, ICAM, MCP, and IL-6 transcripts were similarly increased in LPS injected Tg26 and wild type mice compared to saline controls. TNFa, IL-10, Interferon-a and b were not increased amongst any of the groups.

Several HIV proteins can alter the ability of cells to respond to inflammatory stimuli. In particular, the HIV proteins Vpr and Tat influence the production of immune modulating cytokines and processing of immunogenic proteins[31], [32]. Since production of proinflammatory mediators by renal parenchymal cells is an important component of the pathogenesis of AKI, we investigated whether expression of these factors is different in Tg26 mice compared to WT mice after LPS or saline injection. Twenty-four hours after control or LPS injection, total kidney RNA was harvested from each mouse and expression of inflammatory mediators was analyzed via qPCR. There were no significant differences between saline-injected WT and Tg26 mice for expression of any of the genes investigated (Table 1). In addition, LPS did not induce significant changes in expression of *TNFa, IFN-a, IFN-b,* or *IL-10* in Tg26 or WT mice compared to saline-injected controls (Table 1). Renal expression of several proinflammatory mediators (*CXCL5, IL-6, ICAM,* and *MCP-1*) was markedly increased by LPS injection in Tg26 and WT mice (Figure 4). However, there were no significant differences in the increases in expression of these inflammatory mediators between LPS-injected Tg26 and WT mice (Table 1).

**Table 1:** qPCR analysis of fold change compared to wild type control injected mice.

	Tg26 Ctrl average fold change	WT LPS average fold change	Tg26 LPS average fold change	Overall p-value	p-value for WT Ctrl vs Tg26 LPS	p-value for WT Ctrl vs WT LPS	p-value for WT Ctrl vs Tg26 Ctrl	p-value for WT LPS vs Tg26 LPS
CXCL5	0.76	9.60	9.84	.002	.004	.002	.661	.833
ICAM	1.65	7.96	5.08	.009	.016	.009	.380	.347
MCP	1.49	8.49	6.56	.008	.005	.008	.464	.347
TNF	0.83	0.94	0.73	.303	^______^	^______^	^______^	^______^
IL6	1.16	5.69	4.90	.032	.068	.032	.770	.883
IL10	0.70	2.02	0.92	.869	^______^	^______^	^______^	^______^
IFN a	0.45	0.60	0.73	.915	^______^	^______^	^______^	^______^
IFN b	0.63	0.44	0.51	.915	^______^	^______^	^______^	^______^

## Discussion

The relationship between AKI and increased mortality has been clearly established and has not improved appreciably in the past several decades; a recent study found a 41.1% mortality rate in hospitalized patients with AKI[33]. HIV-infected patients are predisposed to AKI both in the hospitalized and ambulatory settings[1], [3]. At present, there are no effective treatments available for established AKI and the most efficacious strategies for prevention are minimizing exposure to nephrotoxins and optimizing hemodynamic status in patients at highest risk for AKI[34]. There have been no previous studies using *in vivo* or *in vitro* models to determine the mechanism by which HIV-infection predisposes patients to AKI. We therefore modeled septic AKI both *in vivo* and *in vitro* to determine whether increased systemic levels of LPS increase renal HIV transcription and if the presence of HIV within the kidney is a predisposing factor for more severe AKI, tubular injury, and altered cytokine induction.

There was no significant difference in renal function or tubular injury between Tg26 and wild-type mice when measured at baseline or 24 hours after LPS injection. We chose to perform these studies in young (one month old) mice, before the Tg26 mice would develop decreased GFR and histological renal disease. Since increased age is a risk factor for AKI in humans and there are no published data on whether pediatric HIV infected patients are at increased risk of AKI, it is possible that young Tg26 mice are protected from increased susceptibility to AKI. However, performing these studies in older mice would be problematic as Tg26 mice would have significant baseline CKD, which would likely predispose them to AKI, independent of HIV-specific effects.

The dosage of LPS administered caused detectable changes in SUN for most, but not all mice. It is possible that this dose may have obscured an effect that would have been seen at a lower dosage. However, since LPS does not significantly alter renal HIV expression or AKI-associated gene expression, we consider it unlikely that other dosages of LPS would reveal an increased propensity to AKI in the Tg26 mice.

LPS injection did not induce a significant change in renal HIV RNA compared to saline injected Tg26 controls. Moreover, we found that LPS induced a 60-fold increase in splenic HIV RNA levels. The magnitude of this increase in splenic HIV expression is similar to that reported by Tanaka *et al* [26], supporting the validity of our data obtained using the Tg26 mouse model. Our findings emphasize *in vivo* cell specific differences in HIV expression demonstrated by others *in vitro*.

Previous studies using Tg26 mice have shown that a low level of renal HIV expression is correlated with the level of baseline kidney NF-κB activation. Conversely, in the gut, where baseline NF-κB activation is higher, HIV transcription is increased[35]. Though LPS injection increased expression of several NF-κB regulated genes in the kidneys of Tg26 mice, this increase in NF-κB activity was not accompanied by an increase in HIV transcription. It is possible that LPS injection induced expression of systemic and/or local factors that suppressed the ability of NF-κB to activate HIV expression. Though we did not detect upregulation of obvious candidate suppressors of HIV transcription, such as interferon, it is possible that these mediators may have been upregulated at earlier time points or that other factors prevented LPS-induced HIV transcription.

Martinka et al. demonstrated persistent NF-κB activation in podocytes from Tg26 mice.[36] Though podocytes were clearly present in the Tg26 kidney specimens used in our *in vivo* studies, RTEC constitute the great majority of renal cells. Therefore, no conclusions can be drawn from our data regarding the effect of LPS on HIV expression in podocytes. In addition, we only studied the *acute* effects of large dosages of LPS on RTEC HIV expression whereas chronic exposure to lower dosages of LPS may have different effects upon renal function.

Our *in vitro* studies, were performed to address potential limitations of the HIV-transgenic model. Specifically, murine cells may not accurately model HIV transcriptional regulation due to species-specific factors[27]. For example, Tat, a critical HIV transcription factor, interacts with cyclin T1 (CycT1) at the HIV LTR to promote HIV transcription[37]. However, murine CycT1 is unable to facilitate Tat-mediated transcription[27].

To confirm the importance of our data from the Tg26 mice, we repeated experiments using the human RTEC cell line, RT3. Since human RTEC undergo apoptosis after chronic HIV infection *in vitro* [29], RT3 cells were incubated with LPS four days after HIV transduction, before HIV-induced cytotoxicity is observed. The change in HIV transcription in LPS-exposed RT3 cells even at 48 hours was quite modest and certainly lower than that seen in mouse spleen. Overall, our *in vitro* studies suggest that LPS does not induce a clinically significant increase in RTEC HIV transcription and support our *in vivo* findings.

LPS-induced cytokine production is a critical determinant of AKI in murine models of septic AKI[6], [38], [39]. Because expression of viral proteins can further alter cytokine production[40]–[42], we hypothesized that an exaggerated cytokine response would potentiate the effect of HIV expression in the kidney and worsen the course of LPS-induced AKI in Tg26 mice. Our analysis included cytokines, chemokines, and adhesion molecules known to play important roles in LPS induced kidney injury[6], [38], [39]. Though LPS induced marked increases in the expression of several LPS responsive genes in Tg26 and wild-type mice there were no significant differences between Tg26 and wild-type mice in the magnitude of induction for the inflammatory mediators we examined. While it is possible that we may have detected differences in expression of inflammatory mediators if we had studied earlier or later time points, since there was no difference in severity of renal injury between the LPS injected groups, it is not likely that such differences would have been relevant to the pathogenesis of AKI susceptibility.

In clinical studies, the factor that is most closely associated with increased risk of AKI in HIV-infected patients is the presence of CKD [1]. In a recent case series, Wyatt et al reported that 84% of renal autopsy specimens from patients who died with HIV/AIDS had evidence of histological renal disease, though only one third had clinical evidence of CKD prior to death. It is possible that the high prevalence of “silent” CKD in HIV-infected patients is an important factor that predisposes these patients to AKI.

The Tg26 mice used in these experiments had normal renal function at the time of injection so the combined effects of sepsis, HIV infection, and CKD upon AKI susceptibility cannot be extrapolated from this work. Furthermore, sepsis-related AKI often occurs in the patients with several risk factors including volume depletion, hypotension and nephrotoxin exposure. Our model was not designed to measure the effects of all the factors that may be encountered in clinical practice and it is possible that another model of sepsis-induced AKI may produce alternate results.

In conclusion, LPS-induced activation of renal HIV transcription does not explain the increased susceptibility of HIV-infected patients to AKI. This is the first published study examining the pathomechanisms underlying AKI in the setting of HIV infection. Additional research is needed to identify the factors responsible for increased AKI in HIV-infected patients to allow development of improved strategies for the care of this vulnerable patient population.
